# Exposure to pesticides and the risk of hypothyroidism: a systematic review and meta-analysis

**DOI:** 10.1186/s12889-023-16721-5

**Published:** 2023-09-26

**Authors:** Wachiranun Sirikul, Ratana Sapbamrer

**Affiliations:** Department of Community Medicine, Faculty of Medicine, Chiang Mai University, 110 Inthavaroros Road, Sri Phum Subdistrict, Muang District, Chiang Mai, 50200 Thailand

**Keywords:** Pesticides, Hypothyroidism, Thyroid diseases, Insecticides, Herbicides, Fungicides

## Abstract

**Background:**

Knowledge surrounding the association between exposure to pesticides and hypothyroidism is inconsistent and controversial.

**Methods:**

The aim of present study was, therefore, to review scientific evidence systematically and conduct a meta-analysis into the contribution of exposure to pesticides to hypothyroidism. PubMed, Scopus, Web of Science, and Google Scholar were searched. The findings are presented as OR, HR, PR, IRR, and 95% confidence interval (95%CI). A fixed-effect model using the inverse-variance method and random-effects inverse-variance model with DerSimonian-Laird method were used for estimating the pooled estimates. Cochran Q and I^2^ tests were used to confirm the heterogeneity of selected studies.

**Results:**

Twelve studies were included in the systematic review, and 9 studies in the meta-analysis. Epidemiological evidence suggested that exposure to insecticides including organochlorines, organophosphates, and pyrethroids increased risk of hypothyroidism (adjusted odds ratio (aOR) = 1.23, 95%CI = 1.14, 1.33 for organochlorines, aOR = 1.12, 95%CI = 1.07, 1.17 for organophosphates, aOR = 1.15, 95%CI = 1.03, 1.28 for pyrethroids). Exposure to herbicides also increased risk of hypothyroidism (aOR = 1.06, 95%CI = 1.02, 1.10). However, exposure to fungicides and fumigants was not found to be associated with hypothyroidism.

**Conclusion:**

To increase current knowledge and confirm evidence to date future research needs to center on large-scale longitudinal epidemiological and biological studies, examination of dose–response relationships, the controlling of relevant confounding variables, using standardized and high sensitivity tools, and investigating the effects of environmental exposure.

**Supplementary Information:**

The online version contains supplementary material available at 10.1186/s12889-023-16721-5.

## Background

Thyroid disease is a global health issue that has significant adverse effects on well-being. Hypothyroidism is one example of thyroid disease, the pathology of which is thyroid hormone deficiency. The prevalence of hypothyroidism has been found to range from 0.3–3.7% in the USA and 0.2–5.3% in Europe [1]. Thyroid stimulating hormone (TSH) and free thyroxine (fT4) in serum are used to define thyroid dysfunction. Subclinical hypothyroidism is defined as TSH above the normal range but normal fT4, whereas overt hypothyroidism is defined as TSH above the normal range and fT4 below the normal range [2, 3]. The most common symptoms include weight gain, constipation, fatigue, lethargy, cold intolerance, dry skin, and change in voice. It may have major adverse health consequences and eventually death if left untreated [4, 5]. Risk factors for hypothyroidism include female sex, aging, iodine nutrition, genetic susceptibility, and endocrine disrupting chemicals (EDCs) [4–6].

It has been acknowledged that EDCs can disrupt the endocrine system, including the function of the thyroid. Some pesticides have been reported to act as EDCs and also disrupt thyroid function. EDCs may interfere with thyroid function through several mechanisms, including disruption at the hypothalamus–pituitary–thyroid axis, interference with TSH receptors, inhibition of sodium-iodine symporters, inhibition of the thyroid peroxidase enzyme, alteration of the binding sites of transport proteins, inhibition of deiodinase enzymes, increased synthesis of glucuronosyltransferase, decreased cellular uptake of thyroid hormone, and alteration during the transcription of thyroid hormone receptors [7–11].

Previous experimental studies on animals have shown that organochlorine insecticides can cause a decrease in triiodothyronine (T3), and thyroxine (T4), and an increase in TSH [12–14]. Most epidemiological studies focus on the effects of pesticide exposure on thyroid hormone levels, however, the evidence has shown inconsistent results [14–16]. With regard to epidemiological studies, some found an association between pesticide exposure and increased risk of hypothyroidism, but some studies found no association, hence current available evidence is inconsistent, and there is a lack of clarity in which types of pesticides contribute to hypothyroidism. In an attempt to address these issues, we conducted a systematic review and meta-analysis to summarize the findings and identify which pesticides contribute to hypothyroidism.

## Methods

### Search strategy

The aim of this study was to systematically review scientific evidence and conduct a meta-analysis into the relationship between exposure to pesticides and hypothyroidism. The study was carried out in accordance with the Preferred Reporting Items for Systematic Reviews and Meta-Analysis (PRISMA) [17]. A reference management program (Endnote X9.3.3) was used to search the results. The search process was performed by two reviewers (RS and WS). Full-text articles published in PubMed, Web of Science, Scopus, and Google Scholar were searched using the following keywords: “pesticide” OR “insecticide” OR “herbicide” OR “fungicide” OR “nematocide” OR “fumigants” plus “thyroid” OR “thyroid disease” OR “hypothyroidism”. To avoid unplanned research duplication, the study was registered under International Prospective Register of Systematic Reviews (PROSPERO) (CRD42022342522, 7 July 2022). The search started on July 7, 2022, and completed on August 4, 2022.

The articles that were included qualitative and quantitative synthesis were as follows: (1) original articles; (2) published between 1994 and 2022; (3) published as a full-text article; (4) written in English language and others; (5) hypothyroidism was assessed and diagnosed by a physician or by laboratory results of thyroid hormones; (6) the association between exposure to pesticides and hypothyroidism was assessed; (7) data were analyzed by chi-square and /or regression analysis; and (8) presented results using odds ratio (OR), prevalence ratio (PR), hazard ratio (HR), incidence rate ratio (IRR), or relative risk (RR). The studies that were without variables of interest, were review articles, animal studies, involved pregnancy and neonatal hypothyroidism, and irrelevant information were excluded from the study.

### Exposure and outcome classification

Main exposure: The studies were classified as those investigating: (1) insecticides; (2) herbicides; (3) fungicides; (4) fumigants; or (5) non-specific pesticides. The studies into insecticides were also subdivided into organochlorines, organophosphates, carbamates, and pyrethroids. Exposure assessment was classified as occupational or environmental exposure.

Main outcome: all types of hypothyroidism, including subclinical and overt hypothyroidism were included in the study. Outcome assessment was classified as self-reported history of physician diagnosis or laboratory investigation.

### Data extraction

The data were independently extracted by two investigators (RS and WS). The data extracted were as follows: authors, year of publication, study site, study design, population, sample size, type of hypothyroidism, outcome assessment, source of exposure, exposure assessment, name of pesticides, findings, and confounding variables. The findings are presented as OR, HR, PR, IRR, and 95% confidence interval (95%CI). The data regarding types of pesticides were divided into five groups on the basis of the different types of pesticide, insecticides, herbicides, fungicides, fumigants, and non-specific pesticides. The data for insecticides was also subdivided into organochlorines, organophosphates, carbamates, and pyrethroids.

### Quality assessment

The National Heart, Lung, and Blood Institute (NHLBI): guidelines for reporting observational cohort, cross-sectional, and case–control studies were used to assess the quality of eligible articles [18]. Fourteen items on the NHLBI checklist are for reporting cross-sectional and observational cohort studies, and 12 are for reporting case–control studies. The quality of articles was rated as “good”, “fair” or “poor”. The range of scores for cross-sectional and observational cohort studies was 1–5 as poor, 6–10 as fair, and 11–14 as good. The range of scores for case–control studies was 1- 4 as poor, 5–8 as fair, and 9–12 as good. The quality of eligible articles was independently assessed by two reviewers (RS and WS). The decision regarding the inclusion of the studies in the review was agreed by both reviewers. Table S1 and Table S2 present the quality assessment of eligible studies. Of the 12 eligible studies, 8 were assessed as good, 1 as fair, and 3 as poor.

### Data analysis

To pool the effect estimate by a meta-analysis, the type of effect estimate varied between the included studies in our study, necessitating the conversion of PR, RR, and HR into a common metric (OR). The study which presented the results using PR were converted to OR [19]. Shrestha et al. [20, 21] conducted two cohort studies that provided association estimates as an adjusted hazard ratio (aHR). We planned to convert HR to RR and then convert to OR using the following equations [22, 23]:

1 $$\mathrm{RR}= \frac{1-{\mathrm{e}}^{\mathrm{HR}\times \mathrm{ln}(1-\mathrm{r})}}{\mathrm{r}}$$

2 $$\mathrm{RR}= \frac{\mathrm{OR}}{\left(1-\mathrm{r}\right)+(\mathrm{r}\times \mathrm{OR})}$$

Nevertheless, we were unable to calculate the OR from the available data and have reported it as aHR because the total number of exposure and non-exposure groups, and the incidence rates of hypothyroidism were not reported. Since the incidence of hypothyroidism in the studies by Shrestha et al. [20, 21] were reported as 2.38% and 6.94%, respectively, and the reported aHR was relatively close to 1, the HR, OR, and RR would be relatively similar according to the assumption of rare disease/event (less than 10%). To prove this assumption, we performed the conversion of HR to RR and then convert this to OR using the Eqs. (1) and (2). As shown in Figure S1, we found that the converted OR and HR were relatively similar when the incidence rate is less than 10%. Therefore, we decided to perform a meta-analysis using the aHR from the aforementioned studies. The studies examining the association between exposure to non-specific pesticides and hypothyroidism were excluded from the meta-analysis because the reported estimates in these studies were not adjusted for confounding variables [24–26].

The 9 studies which were eligible were included in the meta-analysis. A fixed-effect model using the inverse-variance method and random-effects inverse-variance model with DerSimonian-Laird method were used for estimating the pooled estimates. A fixed-effect meta-analysis was used to estimate pooled effects under the assumption that the true effect of a pesticide exposure (in both magnitude and direction) is the same in the included studies. This assumption implies that the observed differences among study results are due solely to the chance that there was no statistical heterogeneity (low heterogeneity). When heterogeneity across the included studies was moderate to high and statistically significant, a random-effects analysis was performed to estimate pooled effects under the assumption that the effects estimated in the included studies are not identical but instead follow some distribution. Cochran Q and I^2^ tests were used to confirm the heterogeneity of selected studies. The three criteria of the heterogeneity were as follows: low heterogeneity (I^2^ < 25%); moderate heterogeneity (I^2^ 25–50%); substantial heterogeneity (I^2^ > 50%).

Evaluation of the possible bias from small-study effects (e.g. publication bias) was examined through funnel plot visualization. Log adjusted odds ratio (aOR) of individual studies were plotted on the horizontal axis of the funnel plot, and standard error on the vertical axis. Two-tailed statistical tests at a significance of *p*-value < 0.05 were used. The sensitivity analysis on sources of exposure (occupational and environmental), types of reported estimate (OR and HR) and the impact of fixed-effect or random-effects models on summary measures were performed. The data were analyzed using the STATA software package (Stata Corp. 2019.Stata Statistical Software: Release 16. College Station, TX, USA: Stata Corp LLC.).

## Results

### Search study

**Fig. 1:**
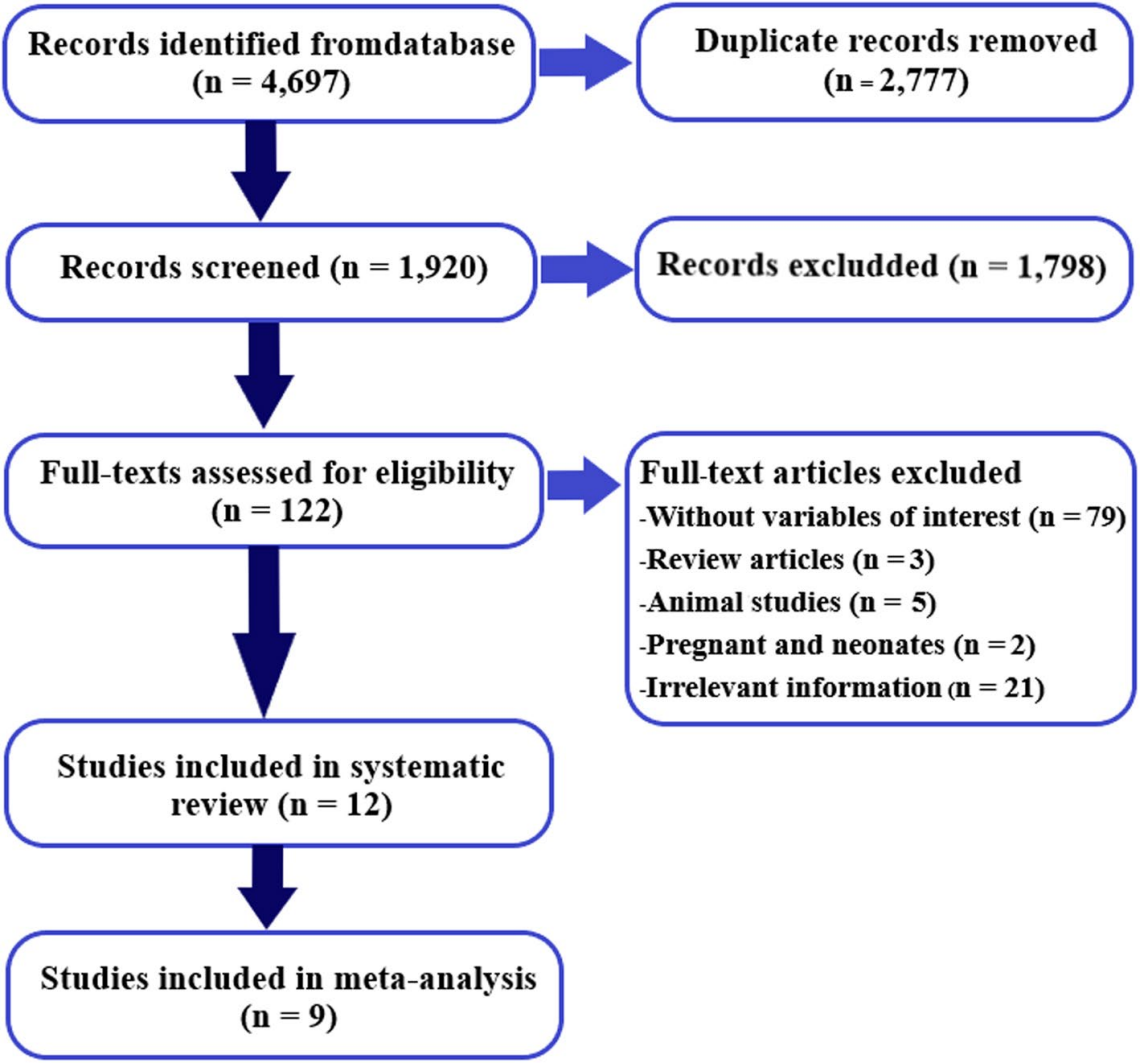
The PRISMA flow diagram of study selection

The PRISMA flow diagram is shown in Fig. 1. The process of study selection was as follows: 4,697 records identified through databases, 1,920 records remained after deletion of duplicates, 122 articles remained after screening for full-text articles, 12 articles were eligible for inclusion in the systematic review, and 9 articles were included in the meta-analysis. One hundred and ten full-text articles (*n* = 110) were excluded due to being without variables of interest (*n* = 79), review articles (*n* = 3), animal studies (*n* = 5), studies in pregnant women and neonates (*n* = 2), and irrelevant information (*n* = 21).

### Association between exposure to insecticides and risk of hypothyroidism

#### Organochlorines (OCs)

Eight studies (*n* = 8) were eligible for inclusion in the qualitative synthesis. Five studies were cohort studies, whereas two were cross-sectional studies, and one was a case–control study [20, 21, 27–32]. Six studies were conducted in the USA (*n* = 6), whereas the others were conducted in Columbia (*n* = 1) and Belgium (*n* = 1). Of the eight studies, six (*n* = 6) found an association between exposure to organochlorine insecticides and risk of hypothyroidism [20, 27–31]. A study by Goldner et al. [27] found an association between hypothyroidism and exposure to organochlorine insecticides (aOR = 1.2, 95%CI = 1.0, 1.6). A study by Goldner et al. [28] also found an association between hypothyroidism and chlordane aOR = 1.36, 95%CI = 1.12, 1.66), heptachlor (aOR = 1.3, 95%CI = 1.04, 1.62), lindane (aOR = 1.35, 95%CI = 1.1, 1.66), and toxaphene (aOR = 1.35, 95%CI = 1.07, 1.7). A study by Wei et al. [29] found an association with 2,5-DCP (adj.OR = 12.86, 95%CI = 1.39, 118.64). Lerro et al. [30] found an association with aldrin (aOR = 4.76, 95%CI = 1.53, 14.8). The study by Shrestha et al. [20] found an association with aldrin for age > 62 years (aHR = 1.28, 95%CI = 1.02, 1.60), chlordane (aHR = 1.21, 95%CI = 1.04,1.41), heptachlor for age > 62 years (aHR = 1.3 5, 95%CI = 1.07, 1.70), and lindane for age > 62 years (aHR = 1.54, 95%CI = 1.23, 1.94). A study by Londoño et al. [31] found an association with Delta-BHC (aOR = 6.8, 95%CI = 1.8, 57.6), endosulfan1 (aOR = 9.9, 95%CI = 1.1, 86.2), and trans-chlordane (aOR = 9.8, 95%CI = 1.1, 86.2) (Table 1).

**Table 1:** Studies regarding exposure to insecticides and hypothyroidism

Authors (year)	Study site/Study design	Population	Sample size	Type of hypothyroidism	Outcome assessment	Source of exposure	Exposure assessment	Types of insecticides	Name of pesticides	Findings OR/HR/PR (95%CI)	Confounding variables
Goldner et al. (2010) [27]	USA/ Cohort study	Female spouses of applicators	15,600 -Hypothyroidism (1,114) -No disease (14,486)	Any hypothyroidism	Self-reported history of physician diagnosis	Occ	Interview	OC	Organochlorines Aldrin Chlordane DDT Heptachlor Lindane	OR = 1.2 (1.0, 1.6)^a^ OR = 1.3 (0.64, 2.4) OR = 1.3 (0.99, 1.7) OR = 1.2 (0.85, 1.6) OR = 1.2 (0.66, 2.3) OR = 1.5 (0.93, 2.4)	Education, age, smoking status, body mass index, hormone replacement therapy
								OP	Organophosphates Chlorpyrifos Coumaphos Diazinon Dichlorvos Fonofos Malathion Phorate Parapthion Terbufos	OR = 1.1 (0.94, 1.2) OR = 1.0 (0.74, 1.4) OR = 1.0 (0.57, 1.9) OR = 1.1 (0.86, 1.3) OR = 0.60 (0.37, 0.97) OR = 0.99 (0.60, 1.6) OR = 1.1 (0.92, 1.3) OR = 0.86 (0.51, 1.4) OR = 1.0(0.53, 2.0) OR = 1.0 (0.70, 1.5)	
								CAR	Carbamates Carbaryl Carbofuran	OR = 1.0(0.88,1.1) OR = 1.0(0.88,1.2) OR = 1.0(0.59,1.6)	
								PYR	Permethrin (crops)	OR = 0.6 (0.4, 1.1)	
Goldner et al. (2013) [28]	USA/ Cohort study	Male pesticide applicators	21,788 -Hypothyroid ism (461) -No disease (21,327)	Any hypothyroidism	Self-reported history of physician diagnosis	Occ	Interview	OC	Aldrin Chlordane DDT Dieldrin Heptachlor Lindane Toxaphene	OR = 1.09 (0.88, 1.36) OR = 1.36 (1.12, 1.66)^a^ OR = 1.25 (1.0, 1.56) OR = 1.04 (0.76, 1.42) OR = 1.3 (1.04, 1.62)^a^ OR = 1.35 (1.1, 1.66)^a^ OR = 1.35 (1.07, 1.7)^a^	Body mass index, age, education
								OP	Chlorpyrifos Coumaphos Diazinon Dichlorvos Fonofos Malathion Parathion Phorate Terbufos Trichlorfon	OR = 1.12 (0.93, 1.34) OR = 1.24 (0.93, 1.67) OR = 1.24 (1.02, 1.5)^a^ OR = 1.26 (0.97, 1.64) OR = 1.08 (0.87,1.33) OR = 1.29 (1.03, 1.62)^a^ OR = 1.2 (0.94,1.52) OR = 1.05 (0.87, 1.27) OR = 1.01 (0.83, 1.22) OR = 2.19 (0.95, 5.03)	
								CAR	Aldicarb Carbaryl Carbofuran	OR = 0.99 (0.72, 1.37) OR = 1.08 (0.89, 1.31) OR = 1.31 (1.08, 1.59)^a^	
								PYR	Permethrin (crops) Permethrin (animals)	OR = 1.19 (0.92, 1.55) OR = 1.14 (0.88, 1.48)	
Wei et al. (2016) [29]	USA/ cross-sectional study	Adolescents (12–19 yrs.)	618 -Hypothyroidism (16) -No disease (602)	Overt hypothyroidism	Laboratory	Env	Blood	OC	2,5-DCP 2,4-DCP	OR = 12.86 (1.39, 118.64)^a^ OR = 0.46 (0.02, 9.41)	Age, sex, race, ethnicity, poverty status, body mass index, physical activity, serum cotinine, urinary iodine, urinary creatinine
Lerro et al. (2017) [30]	USA/ Cohort study	Male pesticide applicators	679 -Hypothyroidism (127) -No disease (552)	Subclinical hypothyroidism	Laboratory	Occ	Interview	OC	Aldrin Chlordane DDT Heptachlor	OR = 4.76 (1.53, 14.8)^a^ OR = 1.80(0.57, 5.73) OR = 0.85(0.26, 2.81) OR = 0.63(0.16, 2.46)	Age, state, body mass index, smoking status, correlated pesticides
								OP	Chlorpyrifos Diazinon Fonofos Malathion Phorate Terbufos	OR = 0.71 (0.36, 1.41) OR = 1.88 (0.68, 5.17) OR = 1.09 (0.52, 2.31) OR = 1.50 (0.69, 3.27) OR = 1.10 (0.47, 2.58) OR = 0.95 (0.51, 1.78)	
								CAR	Carbaryl Carbosulfan	OR = 0.77 (0.27, 2.23) OR = 1.58 (0.81, 3.02)	
								PYR	Permethrin	OR = 1.14(0.54, 2.39)	
Shrestha et al. (2018)^a^ [20]	USA/ Cohort study	Pesticide applicators	34,879 -Hypothyroidism (829) -No disease (34,050)	Any hypothyroidism	Self-reported history of physician diagnosis	Occ	Interview	OC	Aldrin (age ≤ 62) Aldrin (age > 62) Chlordane DDT (age ≤ 62) DDT (age > 62) Dieldrin (age ≤ 62) Dieldrin (age.62) Heptachlor (age ≤ 62) Heptachlor (age > 62) Lindane (age ≤ 62) Lindane (age > 62) Toxaphene (age ≤ 62) Toxaphene (age > 62)	HR = 0.87 (0.65, 1.17) HR = 1.28 (1.02, 1.60)^a^ HR = 1.21 (1.04,1.41)^a^ HR = 0.84 (0.64, 1.12) HR = 1.18 (0.95, 1.47) HR = 0.95 (0.59, 1.51) HR = 1.22 (0.93,1.60) HR = 0.80 (0.58, 1.11) HR = 1.3 5(1.07, 1.70)^a^ HR = 1.01 (0.80, 1.28) HR = 1.54 (1.23, 1.94)^a^ HR = 0.78 (0.56, 1.07) HR = 1.14 (0.89, 1.46)	Sex, education, state, smoking status
								OP	Chlorpyrifos Coumaphos (age ≤ 62) Coumaphos (age > 62) Diazinon Dichlorvos Fonofos Malathion Parathion Phorate Terbufos	HR = 1.02 (0.89, 1.18) HR = 0.88 (0.63, 1.24) HR = 1.44 (1.06, 1.95)^a^ HR = 1.27 (1.10,1.48)^a^ HR = 1.42 (1.17, 1.72)^a^ HR = 1.15(0.97, 1.36) HR = 1.23 (1.04, 1.46)^a^ HR = 1.18 (0.97, 1.42) HR = 1.02 (0.88, 1.19) HR = 1.14 (0.98, 1.32)	
								CAR	Aldicarb Carbaryl Carbofuran	HR = 0.76 (0.58, 1.01) HR = 1.13 (0.97, 1.32) HR = 1.1 2(0.97, 1.31)	
								PYR	Permethrin (animals) Permethrin (crops)	HR = 1.20(0.99, 1.46) HR = 1.19(0.98, 1.46)	
Shrestha et al. (2018)^b^ [21]	USA/ Cohort study	Spouses of private pesticide applicators	23,561 -Hypothyroidism (1,627) -No disease (21,934)	Any hypothyroidism	Self-reported history of physician diagnosis	Occ	Interview	OC	Aldrin Heptachlor	HR = 1.55 (0.88, 2.72) HR = 0.7 7(0.41, 1.47)	Education, state, smoking status, correlated pesticides
								OP	Chlorpyrifos Fonofos Malathion Phorate Terbufos	HR = 1.28 (0.96, 1.71) HR = 0.74 (0.46, 1.20) HR = 1.08 (0.94, 1.24) HR = 0.64 (0.41, 1.01) HR = 0.87 (0.59, 1.30)	
								CAR	Carbaryl Carbofuran	OR = 1.02 (0.90, 1.15) OR = 0.97 (0.62, 1.54)	
Londoño et al. (2018) [31]	Columbia/Cross-sectional study	Agricultural workers	819 -Hypothyroidism (10) -No disease (809)	Overt hypothyroidism	Laboratory	Occ	Blood	OC	Lindane Heptachlor Beta-BHC Delta-BHC Endosulfan1 Cis-chlordane Trans-chlordane Endrin	OR = 0.3 (0.03, 2.1) OR = 1.1 (0.3, 4.0) OR = 0.3 (0.06, 1.3) OR = 6.8 (1.8, 57.6)^a^ OR = 9.9 (1.1, 86.2)^a^ OR = 4.0 (0.5, 32.7) OR = 9.8 (1.1, 86.2)^a^ OR = 0.8(0.2,2.8)	Sex, age, sociodemographic data
				Subclinical hypothyroidism					Lindane Heptachlor Aldrin Beta-BHC Delta-BHC1 Endosulfan1 Cis-chlordane Trans-chlordane 4,4’-DDE Endrin 4,4’-DDT Endosulfan sulfate Methoxichlor	OR = 1.3 (0.7, 2.3) OR = 1.7 (1.0, 3.2)^a^ OR = 1.1 (0.3, 3.7) OR = 1.4 (0.8, 2.5) OR = 2.4 (0.5, 10.8) OR = 6.2 (1.6, 24.8)^a^ OR = 3.1 (1.0, 9.4)^a^ OR = 3.6 (0.7, 17.2) OR = 3.8 (1.6, 9.2)^a^ OR = 1.4 (0.8, 2.5) OR = 2.2 (0.7, 6.3) OR = 2.8 (0.6, 13.3) OR = 2.8 (0.3, 24.4)	
Suhartono et al. (2018) [19]	Indonesia/Cross-sectional study	School-aged children (8–10 yrs.)	66 -Hypothyroidism (24) -No disease (42)	Subclinical hypothyroidism	Laboratory	Env	Interview	OP	Organophosphates	PR = 2.4 (1.4, 4.3)^a^	-
Dufour et al. (2020) [32]	Belgium/Case–control study	Patients diagnosed for autoimmune thyroid pathologies	195 -Hypothyroidism (35) -No disease (160)	Overt hypothyroidism	physician diagnosis	Env	Blood		4,4’-DDT	OR = 4.47 (0.96, 20.8)	Age, sex, body mass index, smoking status, delay between sampling and start of the recruitment

^a^significant association; *2,4-DCP* 2,4-dichlorophenol, *2,5-DCP* 2,5-dichlorophenol, *4,4’-DDE* 4,4’-dichlorodiphenyldichloroethylene, *4,4’-DDT* 4,4’-dichlorodiphenyltrichloroethane, *95%CI* 95% confidence interval, *Beta-BHC* Beta-hexachlorocyclohexane, *CAR* Carbamate insecticides, *DDT* Dichlorodiphenyltrichloroethane, *Delta-BHC* Delta-hexachlorocyclohexane, *Env.* Environmental exposure, *HR* Hazard ratio, *OC* Organochlorine insecticides, *Occ.* Occupational exposure, *OP* Organophosphate insecticides, *OR* Odds ratio, *PR* Prevalence ratio, *PYR* Pyrethroid insecticides

**Fig. 2:**
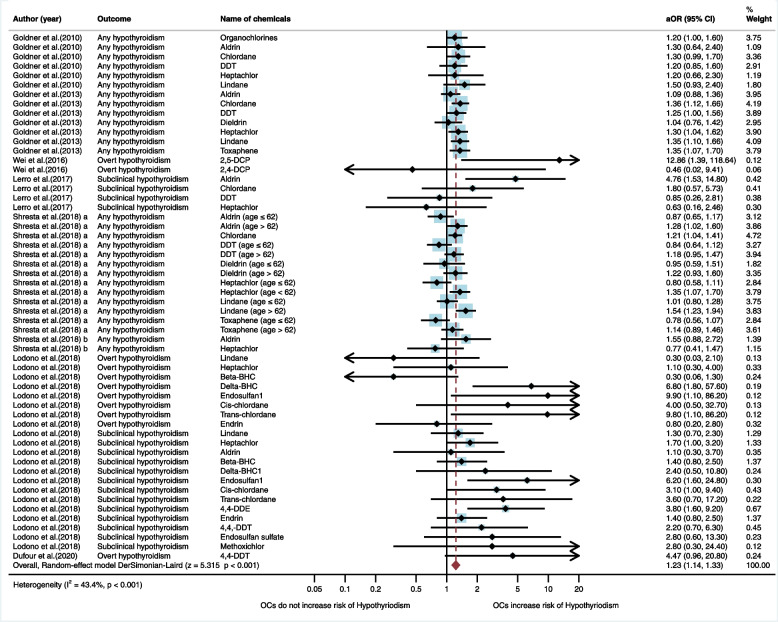
Meta-analysis for exposure to organochlorine insecticides and hypothyroidism

In the meta-analysis for exposure to organochlorines and hypothyroidism, the random-effect model showed that exposure to organochlorines significantly increased the risk of hypothyroidism (aOR = 1.23, 95%CI = 1.14, 1.33, I^2^ = 43.4%, *p* < 0.001) (Fig. 2). The subgroup analysis based on source of exposure, showed that occupational exposure to organochlorines significantly increased the risk of hypothyroidism (aOR = 1.22, 95%CI = 1.14, 1.32, I^2^ = 42.0%, *p* < 0.001), but environmental exposure to organochlorines was not significantly associated with the risk of hypothyroidism (aOR = 4.01, 95%CI = 0.88, 18.23, I^2^ = 32.5%, p = 0.072) (Table S3).

#### Organophosphates (OPs)

**Fig. 3:**
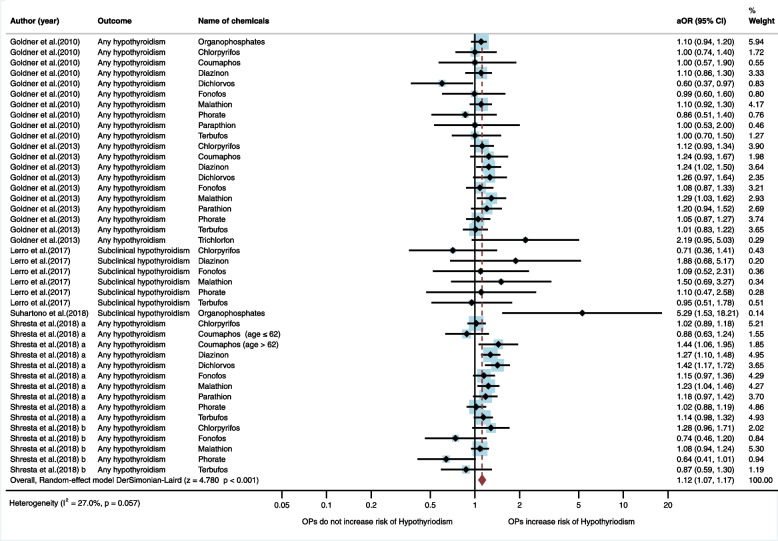
Meta-analysis for exposure to organophosphate insecticides and hypothyroidism

Six studies (*n* = 6) were eligible for inclusion in the qualitative synthesis: five studies were cohort studies, and one was a cross-sectional study [19–21, 27, 28, 30]. Five studies were conducted in the USA (*n* = 5), whereas one study was conducted in Indonesia (*n* = 1). Of the six studies, three studies (*n* = 3) found an association between exposure to organophosphate insecticides and risk of hypothyroidism [19, 20, 28]. A study by Goldner et al. [28] found an association with diazinon (aOR = 1.24, 95%CI = 1.02, 1.5), and malathion (aOR = 1.29, 95%CI = 1.03, 1.62). A study by Suhartono et al. [19] found an association with organophosphate insecticides (crude PR = 2.4, 95%CI = 1.4, 4.3). A study by Shrestha et al. [20] found an association with coumaphos for age > 62 years (aHR = 1.44, 95%CI = 1.06, 1.95), diazinon (aHR = 1.27, 95%CI = 1.10, 1.48), dichlorvos (aHR = 1.42, 95%CI = 1.17, 1.72), and malathion (aHR = 1.23, 95%CI = 1.04, 1.46) (Table 1). In the meta-analysis, the random-effect model showed that exposure to organophosphates significantly increased the risk of hypothyroidism (aOR = 1.12, 95%CI = 1.07, 1.17, I^2^ = 27.0%, *p* < 0.057) (Fig. 3).

#### Carbamates

**Fig. 4:**
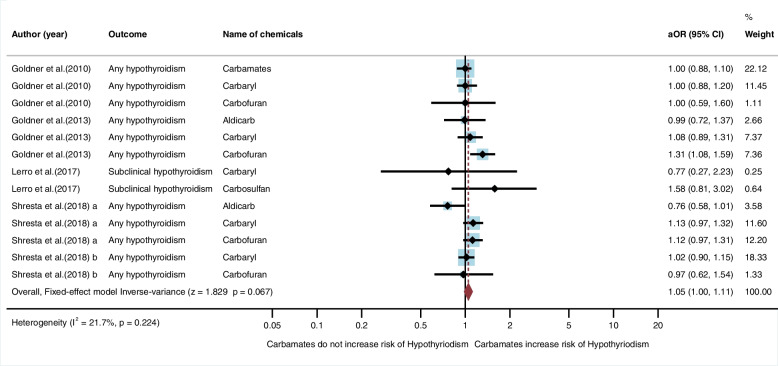
Meta-analysis for exposure to carbamate insecticides and hypothyroidism

Five studies (*n* = 5) were eligible for inclusion in the qualitative synthesis. All studies were cohort studies and conducted in the USA [20, 21, 27, 28, 30]. Of the five studies, only one study found an association between exposure to carbamate insecticides and risk of hypothyroidism. One study by Goldner et al. [28] found an association between exposure to carbofuran and hypothyroidism (aOR = 1.31, 95%CI = 1.08, 1.59) (Table 1). In the meta-analysis, the fixed-effect model showed that exposure to carbamates did not significantly increase the risk of hypothyroidism (aOR = 1.05, 95%CI = 1.00, 1.11, I^2^ = 21.7%, *p* < 0.224) (Fig. 4).

#### Pyrethroids

**Fig. 5:**
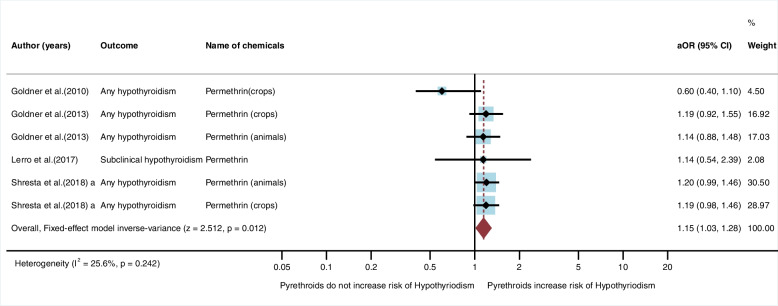
Meta-analysis for exposure to pyrethroid insecticides and hypothyroidism

Four studies (*n* = 4) were eligible for inclusion in the qualitative synthesis. All studies were cohort studies and conducted in the USA [19, 27, 28, 30]. Of the four studies, none found an association between exposure to pyrethroid insecticides and risk of hypothyroidism (Table 1). In the meta-analysis, the fixed-effect model showed that exposure to pyrethroids significantly increased the risk of hypothyroidism (aOR = 1.15, 95%CI = 1.03, 1.28, I^2^ = 25.6%, *p* = 0.242) (Fig. 5).

#### Association between exposure to herbicides and risk of hypothyroidism

**Fig. 6:**
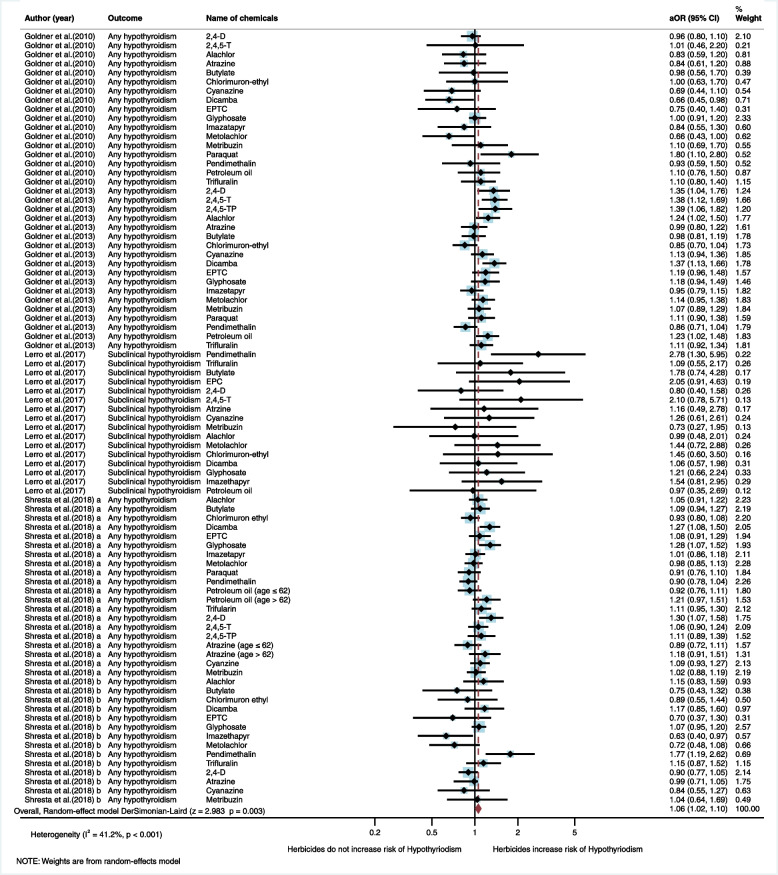
Meta-analysis for exposure to herbicides and hypothyroidism

Five studies (*n* = 5) were eligible for inclusion in the qualitative synthesis. All studies were cohort studies and conducted in the USA, and found an association between exposure to herbicides and risk of hypothyroidism [20, 21, 27, 28, 30]. One study by Goldner et al. [27] found an association with paraquat (aOR = 1.8, 95%CI = 1.1, 2.8). Another study by Goldner et al. [28] found an association between 2,4-D (aOR = OR = 1.35, 95%CI = 1.04, 1.76), 2,4,5-T (aOR = 1.38, 95%CI = 1.12, 1.69), 2,4,5-TP (aOR = 1.39, 95%CI = 1.06, 1.82, alachlor (aOR = 1.24, 95%CI = 1.02, 1.50), dicamba (aOR = 1.37, 95%CI = 1.13, 1.66), and petroleum oil (aOR = 1.23, 95%CI = 1.02, 1.48) and hypothyroidism. The study by Lerro et al. [30] found an association with pendimethalin (aOR = OR = 2.78, 95%CI = 1.30, 5.95). The study by Shrestha et al. [20] found an association with dicamba (aHR = 1.27, 95%CI = 1.08, 1.50), glyphosate (aHR = 1.28, 95%CI = 1.07, 1.52), and 2,4-D (aHR = 1.30, 95%CI = 1.07, 1.58). The study by Shrestha et al. [21] found an association with pendimethalin (aHR = 1.77, 95%CI = 1.19, 2.62) (Table 2). In the meta-analysis, the random-effect model showed that exposure to herbicides significantly increased the risk of hypothyroidism (aOR = 1.06, 95%CI = 1.02, 1.10, I^2^ = 41.2%, *p* < 0.001) (Fig. 6).

**Table 2:** Studies regarding exposure to herbicides and hypothyroidism

Authors (year)	Study site/Study design	Population	Sample size	Type of hypothyroidism	Outcome assessment	Source of exposure	Exposure assessment	Name of pesticides	Findings OR/HR (95%CI)	Confounding variables
Goldner et al. (2010) [27]	USA/ Cohort study	Female spouses of applicators	15,600 -Hypothyroidism (1,114) -No disease (14,486)	Any hypothyroidism	Self-reported history of physician diagnosis	Occ	Interview	2,4-D 2,4,5-T Alachlor Atrazine Butylate Chlorimuron-ethyl Cyanazine Dicamba EPTC Glyphosate Imazatapyr Metolachlor Metribuzin Paraquat Pendimethalin Petroleum oil Trifluralin	OR = 0.96 (0.80, 1.1) OR = 1.01 (0.46, 2.2) OR = 0.83 (0.59, 1.2) OR = 0.84(0.61, 1.2) OR = 0.98 (0.56,1.7) OR = 1.0 (0.63, 1.7) OR = 0.69 (0.44, 1.1) OR = 0.66(0.45, 0.98) OR = 0.75 (0.40, 1.4) OR = 1.0 (0.91, 1.2) OR = 0.84 (0.55, 1.3) OR = 0.66 (0.43, 1.0) OR = 1.1 (0.69, 1.7) OR = 1.8 (1.1, 2.8)^a^ OR = 0.93 (0.59, 1.5) OR = 1.1 (0.76, 1.5) OR = 1.1 (0.80, 1.4)	Education, age, smoking status, body mass index, hormone replacement therapy
Goldner et al. (2013) [28]	USA/ Cohort study	Male pesticide applicators	21,788 -Hypothyroidism (461) -No disease (21,327)	Any hypothyroidism	Self-reported history of physician diagnosis	Occ	Interview	2,4-D 2,4,5-T 2,4,5-TP Alachlor Atrazine Butylate Chlorimuron-ethyl Cyanazine Dicamba EPTC Glyphosate Imazetapyr Metolachlor Metribuzin Paraquat Pendimethalin Petroleum oil Trifluralin	OR = 1.35 (1.04, 1.76)^a^ OR = 1.38 (1.12, 1.69)^a^ OR = 1.39 (1.06, 1.82)^a^ OR = 1.24 (1.02, 1.50)^a^ OR = 0.99 (0.80, 1.22) OR = 0.98 (0.81, 1.19) OR = 0.85 (0.70, 1.04) OR = 1.13 (0.94, 1.36) OR = 1.37 (1.13, 1.66)^a^ OR = 1.19 (0.96, 1.48) OR = 1.18 (0.94, 1.49) OR = 0.95 (0.79, 1.15) OR = 1.14(0.95, 1.38) OR = 1.07 (0.89, 1.29) OR = 1.11 (0.9, 1.38) OR = 0.86 (0.71,1.04) OR = 1.23 (1.02, 1.48)^a^ OR = 1.11 (0.92, 1.34)	Body mass index, age, education
Lerro et al. (2017) [30]	USA/ Cohort study	Male pesticide applicators	679 -Hypothyroidism (127) -No disease (552)	Subclinical hypothyroidism	Laboratory	Occ	Interview	Pendimethalin Trifluralin Butylate EPC 2,4-D 2,4,5-T Atrazine Cyanazine Metribuzin Alachlor Metolachlor Chlorimuron-ethyl Dicamba Glyphosate Imazethapyr Petroleum oil	OR = 2.78 (1.30, 5.95)^a^ OR = 1.09 (0.55, 2.17) OR = 1.78 (0.74, 4.28) OR = 2.05 (0.91, 4.63) OR = 0.80 (0.40, 1.58) OR = 2.10 (0.78, 5.71) OR = 1.16 (0.49, 2.78) OR = 1.26 (0.61, 2.61) OR = 0.73 (0.27, 1.95) OR = 0.99 (0.48, 2.01) OR = 1.44 ( 0.72, 2.88) OR = 1.45 (0.60, 3.50) OR = 1.06 (0.57, 1.98) OR = 1.21 (0.66, 2.24) OR = 1.54 (0.81, 2.95) OR = 0.97 (0.35, 2.69)	Age, state, body mass index, smoking status, correlated pesticides
Shrestha et al. (2018)^a^ [20]	USA/ Cohort study	Pesticide applicators	34,879 -Hypothyroidism (829) -No disease (34,050)	Any hypothyroidism	Self-reported history of physician diagnosis	Occ	Interview	Alachlor Butylate Chlorimuron ethyl Dicamba EPTC Glyphosate Imazetapyr Metolachlor Paraquat Pendimethalin Petroleum oil (age ≤ 62) Petroleum oil (age > 62) Trifularin 2,4-D 2,4,5-T 2,4,5-TP Atrazine (age ≤ 62) Atrazine (age > 62) Cyanzine Metribuzin	HR = 1.05(0.91,1.22) HR = 1.09(0.94,1.27) HR = 0.93(0.80,1.08) HR = 1.27(1.08,1.50)^a^ HR = 1.08(0.91,1.29) HR = 1.28(1.07,1.52)^a^ HR = 1.01(0.86,1.18) HR = 0.98(0.85,1.13) HR = 0.91(0.76,1.10) HR = 0.90(0.78,1.04) HR = 0.92(0.76,1.11) HR = 1.21(0.97,1.51) HR = 1.11(0.95,1.30) HR = 1.30(1.07,1.58)^a^ HR = 1.06(0.90,1.24) HR = 1.11(0.89,1.39) HR = 0.89(0.72,1.11) HR = 1.18(0.91,1.51) HR = 1.09(0.93,1.27) HR = 1.02(0.88,1.19)	Sex, education, state, smoking status
Shrestha et al. (2018)^b^ [21]	USA/ Cohort study	Spouses of private pesticide applicators	23,561 -Hypothyroidism (1,627) -No disease (21,934)	Any hypothyroidism	Self-reported history of physician diagnosis	Occ	Interview	Alachlor Butylate Chlorimuron ethyl Dicamba EPTC Glyphosate Imazethapyr Metolachlor Pendimethalin Trifluralin 2,4-D Atrazine Cyanazine Metribuzin	HR = 1.15(0.83,1.59) HR = 0.75(0.43,1.32) HR = 0.89(0.55,1.44) HR = 1.17(0.85,1.60) HR = 0.70(0.37,1.30) HR = 1.07(0.95,1.20) HR = 0.63(0.40,0.97) HR = 0.72(0.48,1.08) HR = 1.77(1.19,2.62)^a^ HR = 1.15(0.87,1.52) HR = 0.90(0.77,1.05) HR = 0.99(0.71,1.05) HR = 0.84(0.55,1.27) HR = 1.04(0.64,1.69)	Education, state, smoking status, correlated pesticides

^a^significant association; *2,4-D* 2,4-dichlorophenoxyacetic acid, *2,4,5-T* 2,4,5-trichlorophenoxyacetic acid, *2,4,5-TP* 2(2,4,5-trichlorophenoxy) propionic acid, *95%CI* 95% confidence interval, *Env.* Environmental exposure, *EPTC* S-ethyl dipropylthiocarbamate, *HR* Hazard ratio, *Occ.* Occupational exposure, *OR* Odds ratio

#### Association between exposure to fungicides and risk of hypothyroidism

**Fig. 7:**
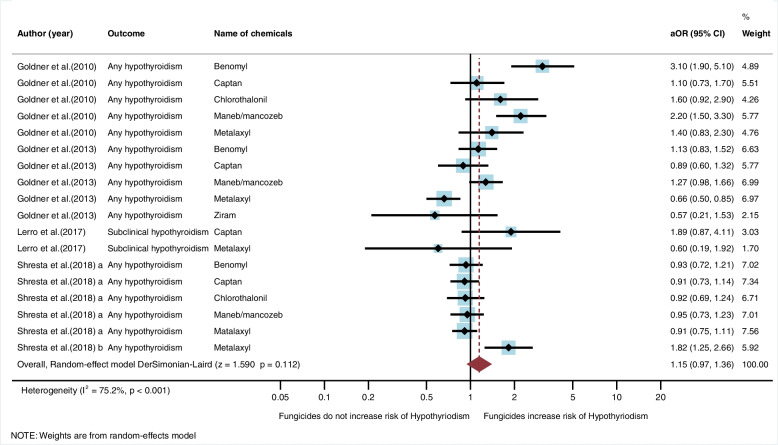
Meta-analysis for exposure to fungicides and hypothyroidism

Five studies (*n* = 5) were eligible for inclusion in the qualitative synthesis. All these were cohort studies and conducted in the USA [20, 21, 27, 28, 30]. Of the five studies, two studies found an association between exposure to fungicides and risk of hypothyroidism [21, 27]. A study by Goldner et al. [27] found an association with benomyl (aOR = 3.1, 95%CI = 1.9, 5.1), and maneb/mancozeb (aOR = 2.2, 95%CI = 1.5, 3.3). The study by Shrestha et al. [21] found an association with metalaxyl (aHR = 1.82, 95%CI = 1.25, 2.66) (Table 3). In the meta-analysis, the random-effect model showed that exposure to fungicides did not significantly increase the risk of hypothyroidism (aOR = 1.15, 95%CI = 0.97, 1.36, I^2^ = 75.2%, *p* < 0.001) (Fig. 7).

**Table 3:** Studies regarding exposure to fungicides and hypothyroidism

Authors (year)	Study site/Study design	Population	Sample size	Type of hypothyroidism	Outcome assessment	Source of exposure	Exposure assessment	Name of pesticides	Findings OR/HR (95%CI)	Confounding variables
Goldner et al. (2010) [27]	USA/ Cohort study	Female spouses of pesticide applicators	15,600 -Hypothyroidism (1,114) -No disease (14,486)	Any hypothyroidism	Self-reported history of physician diagnosis	Occ	Interview	Benomyl Captan Chlorothalonil Maneb/mancozeb Metalaxyl	OR = 3.1 (1.9, 5.1)^a^ OR = 1.1 (0.73, 1.7) OR = 1.6 (0.92, 2.9) OR = 2.2 (1.5, 3.3)^a^ OR = 1.4 (0.83, 2.3)	Education, age, smoking status, body mass index, hormone replacement therapy
Goldner et al. (2013) [28]	USA/ Cohort study	Male esticide applicators	21,788 -Hypothyroidism (461) -No disease (21,327)	Any hypothyroidism	Self-reported history of physician diagnosis	Occ	Interview	Benomyl Captan Maneb/mancozeb Metalaxyl Ziram	OR = 1.1 3(0.83, 1.52) OR = 0.89 (0.6, 1.32) OR = 1.27 (0.98, 1.66) OR = 0.66 (0.5, 0.85) OR = 0.57(0.21, 1.53)	Body mass index, age, education
Lerro et al. (2017) [30]	USA/ Cohort study	Male pesticide applicators	679 -Hypothyroidism (127) -No disease (552)	Subclinical hypothyroidism	Laboratory	Occ	Interview	Captan Metalaxyl	OR = 1.89 (0.87, 4.11) OR = 0.60 (0.19, 1.92)	Age, state, body mass index, smoking status, correlated pesticides
Shrestha et al. (2018)^a^ [20]	USA/ Cohort study	Pesticide applicators	34,879 -Hypothyroidism (829) -No disease (34,050)	Any hypothyroidism	Self-reported history of physician diagnosis	Occ	Interview	Benomyl Captan Chlorothalonil Maneb/mancozeb Matalaxyl	HR = 0.93 (0.72,1.21) HR = 0.91 (0.73, 1.14) HR = 0.92 (0.69, 1.24) HR = 0.95 (0.73, 1.23) HR = 0.91 (0.75, 1.11)	Sex, education, state, smoking status
Shrestha et al. (2018)^b^ [21]	USA/ Cohort study	Spouses of private pesticide applicators	23,561 -Hypothyroidism (1,627) -No disease (21,934)	Any hypothyroidism	Self-reported history of physician diagnosis	Occ	Interview	Metalaxyl	HR = 1.82 (1.25, 2.66)^a^	Education, state, smoking status, correlated pesticides

^a^significant association; *95%CI* 95% confidence interval, *Env.* Environmental exposure, *HR* Hazard ratio, *Occ.* Occupational exposure, *OR* Odds ratio

#### Association between exposure to fumigants and risk of hypothyroidism

**Fig. 8:**
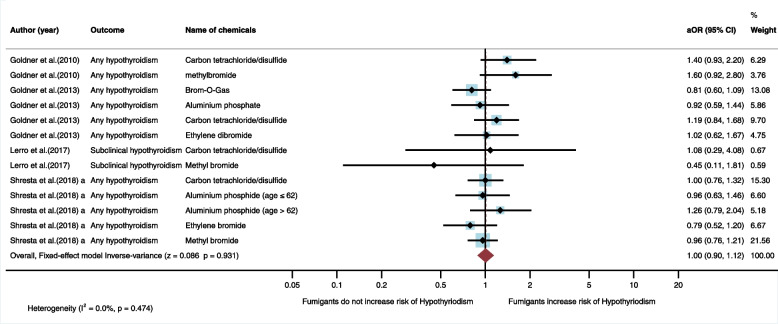
Meta-analysis for exposure to fumigants and hypothyroidism

Four studies (*n* = 4) were eligible for inclusion in the qualitative synthesis. All were cohort studies and conducted in the USA [20, 27, 28, 30]. Of the four studies, none found any association between exposure to fumigants and risk of hypothyroidism (Table 4). In the meta-analysis, the fixed-effect model also showed that exposure to fumigants did not significantly increase the risk of hypothyroidism (aOR = 1.00, 95%CI = 0.90, 1.12, I_2_ = 0%, *p* < 0.474) (Fig. 8).

**Table 4:** Studies regarding exposure to fumigants and hypothyroidism

Authors (year)	Study site/Study design	Population	Sample size	Type of hypothyroidism	Outcome assessment	Source of exposure	Exposure assessment	Name of pesticides	Findings OR/HR (95%CI)	Confounding variables
Goldner et al. (2010) [27]	USA/ Cohort study	Female spouses of pesticide applicators	15,600 -Hypothyroid ism (1,114) -No disease (14,486)	Any hypothyroidism	Self-reported history of physician diagnosis	Occ	Interview	Carbon tetrachloride/carbon disulfide Methylbromide	OR = 1.4 (0.93, 2.2) OR = 1.6 (0.92,2.8)	Education, age, smoking status, body mass index, hormone replacement therapy
Goldner et al. (2013) [28]	USA/ Cohort study	Male esticide applicators	21,788 -Hypothyroidism (461) -No disease (21,327)	Any hypothyroidism	Self-reported history of physician diagnosis	Occ	Interview	Brom-O-Gas Aluminium phosphate Carbon tetrachloride/carbon disulfide Ethylene dibromide	OR = 0.81 (0.6, 1.09) OR = 0.92 (0.59, 1.44) OR = 1.19 (0.84, 1.68) OR = 1.02 (0.62, 1.67)	Body mass index, age, education
Lerro et al. (2017) [30]	USA/ Cohort study	Male pesticide applicators	679 -Hypothyroidism (127) -No disease (552)	Subclinical hypothyroidism	Laboratory	Occ	Interview	Carbon tetrachloride/carbon disulfide Methyl bromide	OR = 1.08 (0.29, 4.08) OR = 0.45 (0.11, 1.81)	Age, state, body mass index, smoking status, correlated pesticides
Shrestha et al. (2018)^a^ [20]	USA/ Cohort study	Pesticide applicators	34,879 -Hypothyroidism (829) -No disease (34,050)	Any hypothyroidism	Self-reported history of physician diagnosis	Occ	Interview	Carbon tetrachloride/carbon disulfide Aluminium phosphide (age ≤ 62) Aluminium phosphide (age > 62) Ethylene bromide Methyl bromide	HR = 1.00 (0.76,1.32) HR = 0.96 (0.63, 1.46) HR = 1.26(0.79, 2.04) HR = 0.79 (0.52, 1.20) HR = 0.96(0.76, 1.21)	Sex, education, state, smoking status

*95%CI* 95% confidence interval, *HR* Hazard ratio, *OCC* Occupational exposure,* OR* Odds ratio

#### Association between exposure to non-specific pesticides and risk of hypothyroidism

Three studies (*n* = 3) were eligible for inclusion in the qualitative synthesis: two studies were cross-sectional studies, and one was a cohort study [24–26]. Of the three studies, two (*n* = 2) found an association between exposure to non-specific pesticides and risk of hypothyroidism [25, 26]. The study by Huang et al. [25] found an association between exposure to non-specific pesticides and hypothyroidism (IRR = 1.40, 95%CI = 1.07, 1.83). The study by Risal et al. [26] also found an association between exposure to non-specific pesticides and hypothyroidism (crude OR = 2.18, 95%CI = 1.31, 3.63) (Table 5).

**Table 5:** Studies regarding exposure to non-specific pesticides and hypothyroidism

Authors (year)	Study site/Study design	Population	Sample size	Type of hypothyroidism	Outcome assessment	Source of exposure	Exposure assessment	Findings OR/PR/IRR (95%CI)	Confounding variables
Kartini et al. (2018) [24]	Indonesia/ Cross-sectional study	Elementary school children (9–12 yrs.)	100	Subclinical hypothyroidism	Laboratory	Env	Interview	PR = 2.2(0.8,6.0)	-
Huang et al. (2017) [25]	Taiwan/Cohort study	Nationwide population	41,488 -Pesticide poisoning (10,372) -No pesticide poisoning (31,116)	Any hypothyroidism	Physician diagnosis	Env	Interview	IRR = 1.40(1.07,1.83)^a^	-
Risal et al. (2019) [26]	Nepal/Retrospective cross-sectional study	Patients	288 -Hypothyroidism (116) -No disease (172)	Any hypothyroidism	Laboratory	Env	Interview	OR = 2.18 (1.31, 3.63)^a^	-

^a^significant association; *95%CI* 95% confidence interval, *Env.* Environmental exposure, *HR* Hazard ratio, *IRR* Incidence rate ratio, *Occ.* Occupational exposure, *OR* Odds ratio, *PR* Prevalence ratio

#### Funnel plots

**Fig. 9:**
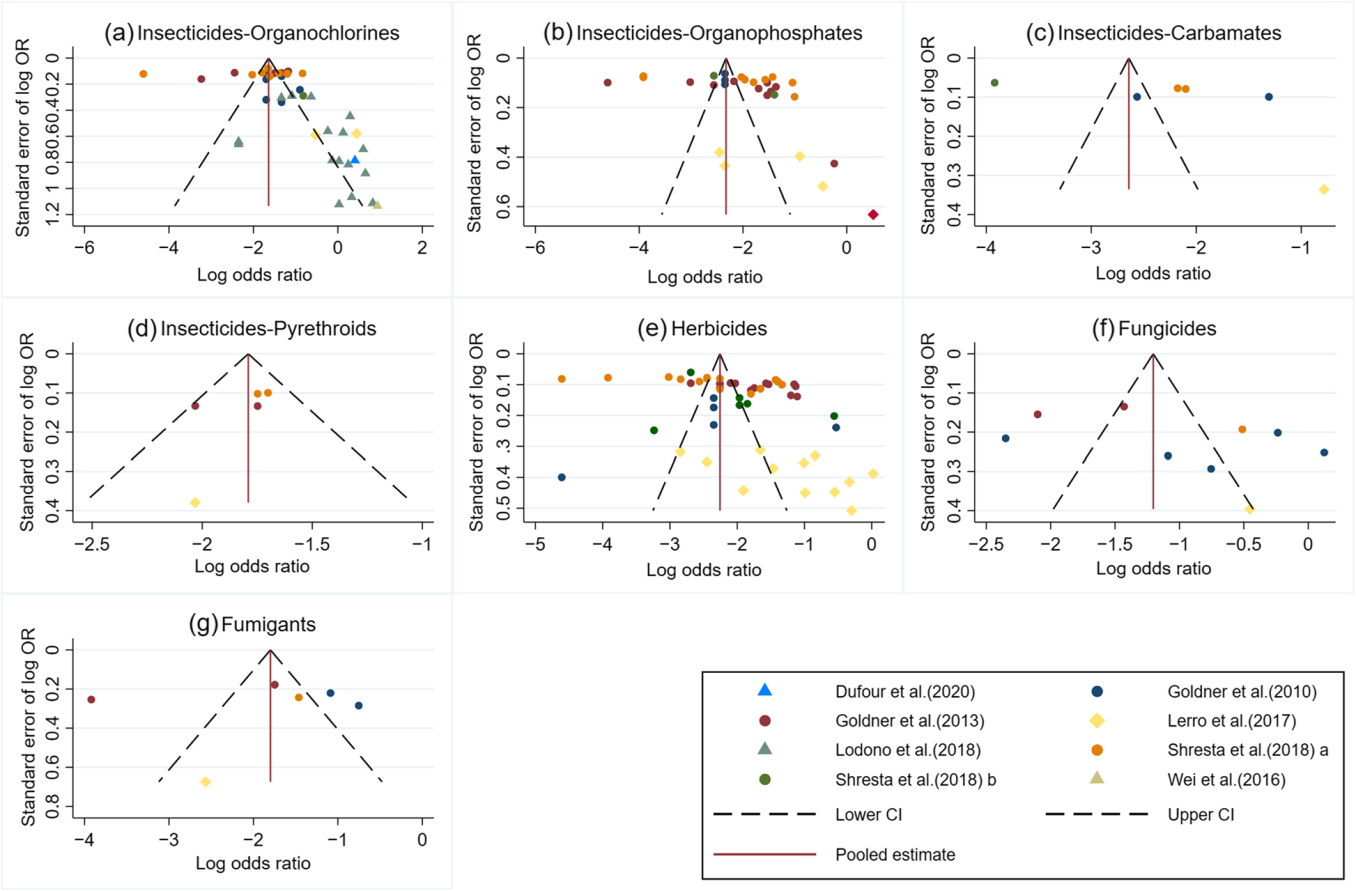
Funnel plot

Funnel plot asymmetries, indicating the evidence of small study effects, were observed in the meta-analyses of all pesticide groups (Fig. 9). When sensitivity analyses were performed, modest changes in effect estimates were found across all outcomes of interest, indicating the robustness of the overall findings (Table S3).

## Discussion

The results from the meta-analysis provided evidence that exposure to insecticides, which included organochlorines, organophosphates, and pyrethroids, significantly increased the risk of hypothyroidism (aOR = 1.23, 95%CI = 1.14, 1.33 for organochlorines, aOR = 1.12, 95%CI = 1.07, 1.17 for organophosphates, aOR = 1.15, 95%CI = 1.03, 1.28 for pyrethroids). Organochlorines have been shown to interfere with synthesis, transportation, and metabolism of thyroid hormones through a variety of mechanisms [8]. Several organochlorines have the ability to mimic thryroid hormones, and bind to thyroid receptors along the hypothalamus-pituitary-thyroid (HPT) axis, resulting in reduced bioactivity of thyroid hormones, such as T3 and T4. The reduced bioactivity of thyroid hormones may occur through the reduction of transport proteins and/or increased clearance of thyroid hormones [7]. An in vivo study found that exposure to DDT in male rats caused decreased total thyroxine (TT4) and fT4 levels, and decreased the levels of transthyretin protein responsible for T4 transport. It also caused increased T4 clearance by upregulating hepatic enzymes [33].

Thyroid disruption may be caused by inhibition of TSH receptors. In vitro studies showed that DDT can inhibit TSH receptors, resulting in decreased production of T4 and T3 levels [8, 34]. Due to the high lipophilic property of DDT, it has the potential to change the phospholipid composition of thyroid cell membranes, and induce the formation of extracellular vesicles containing the TSH receptors. Consequencely, DDT could lead to the triggering of autoimmunity against TSH receptors and induce the failure of TSH receptors [34]. An in vivo study by Yaglova and Yaglov [35] also suggested that long-term exposure to DDT in rats changed the cytophysiology in the follicular epithelium of the thyroid gland, leading to a decrease in the production of thyroid hormones in rats. Some organochlorines can interfere with thyroid hormones by disruption of the HPT axis. In an in vivo study, HCB was shown to disrupt the HPT axis, resulting in decreased T4 levels, increased T4 conversion to T3, and increased inhibition of T4 binding to transporters [36]. In addition, HCB was shown to inhibit cell growth in thyrocytes by inhibiting cell progression of FRTL-5 rat thyroid cells and increasing mRNA levels of transforming growth factor-beta (TGF-β1) [37].

The results from the meta-analysis also provided evidence that occupational exposure to organochlorines significantly increased the risk of hypothyroidism, but no association was found between environmental exposure and hypothyroidism. Occupational exposure to pesticides generally occurs in agricultural workers and workers in pesticide factories. Acute exposure generally occurs in the workers who mix, load, spray, and apply pesticides, whereas long-term exposure occurs in the workers in other tasks. Environmental exposure occurs in the general population through eating foods and drinks contaminated with pesticides, inhaling air contaminated with pesticides, and living close to a contaminated area. Environmental exposure usually results in low dose expose to pesticides, and causes chronic adverse effects [38]. It is likely that those experiencing occupational exposure are exposed to higher doses of pesticides. As a result, the association between organochlorine exposure and hypothyroidism was found only in occupational exposure. The findings of the meta-analysis indicate that pesticides should be used with caution, especially pesticides used occupationally with high doses and long-term exposure.

Regarding organophosphates, previous studies clearly stated that the main mechanism of thyroid disruption is interference with thyrocyte growth [7, 39]. Both in vivo and in vitro studies by Porreca et al. [39] suggested that exposure to chlorpyrifos had an effect on the thyroid system by altering the growth of thyrocytes and decreasing gene expression, leading to decreased thyroid hormone levels. Other mechanisms are decreased gene expression and decreased TSH receptor expression. An in vitro study by Xiong et al. [40] also found the indication that malathion interferes with thyroid function through down-regulation of TSH receptors and cellular cAMP, resulting in suppression of TSH dependent signal transduction, inhibition of thyroid transcription, and inhibition of thyroid hormone biosynthesis. In vitro study by Yang et al. [41] also found that potentially, triazophos and their metabolites could disrupt thyroid hormone receptors, and inhibit binding and transport of thyroid hormones in the blood stream. With regard to pyrethroids, previous in vivo and silico studies found clear evidence that permethrin, bifenthrin, and lampda-cyhalothrin could disrupt thyroid function through binding with tranthyretin receptor proteins, and consequently decreased bioactivity of thyroid hormone levels [42, 43]. In addition, several types of pyrethroid including cyhalothrin, cyfluthrin, cycloprothrin, cypermethrin, deltamethrin, etofenprox, fenvalerate, permethrin, and tetramethrin, could bind with thyroid hormone receptors and show agonist effects [44, 45].

The results of the meta-analysis also provided evidence that exposure to herbicides significantly increased the risk of hypothyroidism (aOR = 1.06, 95%CI = 1.02, 1.10). Amitrole has been shown to disrupt thyroid function through inhibiting the production of thyroid peroxidase in thyroid follicles, resulting in deceased synthesis of T3 and T4 levels. Acetochlor has been also shown to disrupt thyroid function through enhancing the hepatic metabolism and altering mRNA expression of HPT axis-related genes, resulting in increased biliary metabolism of T3 and T4, and decreased thyroid hormone levels in the bloodstream [8, 46]. Pertinent to glyphosate, a review by Romano et al. [47] stated that glyphosate might disrupt thyroid function through iodide oxidation and oxidative phosphorylation in adenosine triphosphate (ATP) synthesis. Nevertheless, few studies into herbicides and thyroid toxicology have been carried out to date. Significantly, our study could not subdivide herbicides based on chemical structure because of the small number of epidemiological studies on herbicides as well as several types of chemical structures. Therefore, it is rather difficult to confirm the evidence based on the formulae of herbicides.

The relevant laboratory studies unearthed by our search provided some information to explain the mechanisms associated with thyroid disruption, and facilitated the interpretation of the epidemiological effects of hypothyroidism [48]. Nevertheless, transference of laboratory data from animal studies to humans is challenging because the mechanisms of action of pesticides may differ between species, and duration and levels of exposure. Synergistic effects and mode of action of pesticide mixtures should be also considered [11]. In addition, conclusive evidence in animal studies is typically only found to affect the thyroid following high doses of pesticide exposure, and the doses found in these animal studies exceed the legal levels of pesticides permitted [14].

This study is the first systematic review and meta-analysis investigating the association between pesticide exposure and the risk of hypothyroidism. Although the epidemiological evidence supported that exposure to insecticides and herbicides contributed to hypothyroidism, some limitations should be considered. Firstly, the majority of studies assessed occupational exposure and studies investigating environmental exposure were limited. Therefore, it is difficult to confirm the evidence based on environmental exposure. Secondly, some studies used interviewing as a tool for assessing exposure to pesticides and outcome. Data collection by interview with regard to pesticide use may be subjective, cannot identify the degree or level of pesticide exposure and may be subjected to recall bias. Therefore, further studies determining the dose–response relationships are warranted. In addition, self-reported histories of physician diagnosis in some studies are unable to identify the types of hypothyroidism. Thirdly, most studies were conducted in the USA; therefore, the evidence might not be generalized to populations in other countries. Although our search terms were broad and not restricted to specific study designs or countries, it is possible that our study might have missed some relevant studies which are only available in non-English language versions or unpublished. Studies reporting a negative effect of the pesticide on hypothyroidism may be published more frequently than studies with null results [49, 50]. Due to the marginal effect of pesticides on hypothyroidism, a large sample size is necessary to determine statistical significance. This could explain why there are very few published studies. However, the sensitivity analysis revealed no or minimal variation in effect estimates, supporting the robustness of the results. Publication bias may have less of an effect on the validity of the current systematic review and meta-analysis [51, 52]. Fourthly, human beings are usually exposed to a mixture of pesticides at the same time, therefore, it is difficult to separate the effect of individual pesticides [11, 53]. Furthermore, some studies investigated only a few types of pesticides, whereas some investigated non-specific pesticides. As a result, over- or under-estimation might have occurred. Fifthly, some studies assessed exposure to pesticides and hypothyroidism by using cross-sectional study. A cross-sectional study design cannot determine cause-and-effect relationships [54]. Sixthly, high heterogeneity was found in some groups, which might be due to different study populations and methodologies, especially as regards study designs, types of exposure, outcome measurement, and effect estimates. The high heterogeneity might have effects on the results, particularly in the meta-analyses carried out into fungicides, herbicides, and organochlorines. Finally, some studies assessed the association between pesticide exposure and hypothyroidism without adjusting covariates for statistical analysis. The adjustment for covariates is to improve the efficiency of the analysis and give more precise evidence. The important covariates contributing to hypothyroidism which need to be considered in further studies include age, gender, race, ethnicity, education, smoking status, body mass index, nutritional status, iodine uptake, poverty status, physical activity, and hormone replacement therapy, investigating the correlation of these to pesticide exposure.

## Conclusion

There is considerable evidence to indicate that exposure to the insecticides organochlorines, organophosphates, and pyrethroids increased risk of hypothyroidism. Exposure to herbicides also increased risk of hypothyroidism. However, exposure to fungicides and fumigants was not found to be associated with hypothyroidism. With regard to the source of exposure, the evidence showed that occupational exposure to organochlorines contributed to hypothyroidism. However, the small number of studies in environmental exposure meant that it is a challenge to draw firm conclusions. To add weight to the conclusions to date in future research, conducting large-scale longitudinal epidemiological and biological studies, examining dose–response relationships, controlling relevant confounding variables, using standardized and high sensitivity tools, and investigating in effects of environmental exposure, are needed to confirm the evidence.

### Supplementary Information

**Additional file 1: Figure S1.** The relationship between Hazard ratios and converted Odds ratios.

**Additional file 2: Table S1.** Quality assessment for reporting observational cohort and cross-sectional, According to the guideline of National Heart, Lung, and Blood Institute (NHLBI). **Table S2.** Quality assessment for reporting case-control study, According to the guideline of National Heart, Lung, and Blood Institute (NHLBI). **Table S3.** Subgroup and sensitivity analyses.

## Data Availability

The data used in the study can be made available from Ratana Sapbamrer (corresponding author) on reasonable request.

## Abbreviations

2,4,5-T: 2,4,5-Trichlorophenoxyacetic acid
2,4,5-TP: 2(2,4,5-Trichlorophenoxy) propionic acid
2,4-D: 2,4-Dichlorophenoxyacetic acid
2,4-DCP: 2,4-Dichlorophenol
4,4’-DDE: 4,4’-Dichlorodiphenyldichloroethylene
4,4’-DDT: 4,4’-Dichlorodiphenyltrichloroethane
95%CI: 95% Confidence interval
aOR: Adjusted odds ratio
ATP: Adenosine triphosphate
Beta-BHC: Beta-hexachlorocyclohexane
CAR: Carbamate insecticides
DDT: Dichlorodiphenyltrichloroethane
Delta-BHC: Delta-hexachlorocyclohexane
Env.: Environmental exposure
EPTC: S-ethyl dipropylthiocarbamate
fT4: Free thyroxine
HR: Hazard ratio
IRR: Incidence rate ratio
OC: Organochlorine insecticides
Occ.: Occupational exposure
OP: Organophosphate insecticides;
OR: Odds ratio
PR: Prevalence ratio
PYR: Pyrethroid insecticides
TSH: Thyroid stimulating hormone
EDCs: Endocrine disrupting chemicals
T3: Triiodothyronine
T4: Thyroxine
PRISMA: Preferred Reporting Items for Systematic Reviews and Meta-Analysis
PROSPERO: International Prospective Register of Systematic Reviews
RR: Relative risk
NHLBI: The National Heart, Lung, and Blood Institute
HPT: Hypothalamus-pituitary-thyroid
TT4: Total thyroxine
