# Supplementary figures and images for: Exposure to pesticides and the risk of hypothyroidism: a systematic review and meta-analysis
Source: BMC Public Health. 2023 Sep 26;23:1867. doi: 10.1186/s12889-023-16721-5 (PMC10523800; doi:10.1186/s12889-023-16721-5)

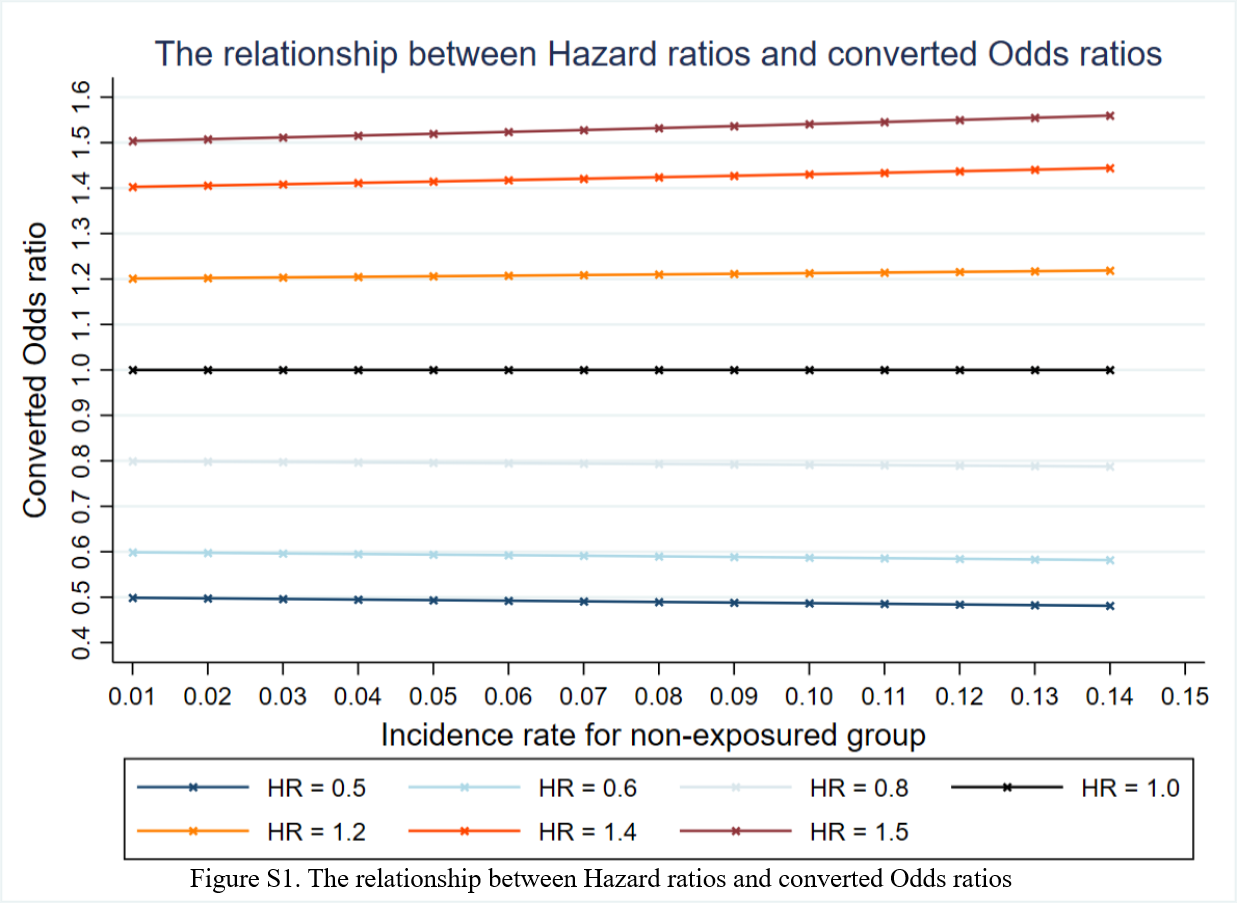

Supplement: Supplementary file 1 — Additional file 1: Figure S1. The relationship between Hazard ratios and converted Odds ratios. [file 12889_2023_16721_MOESM1_ESM.tiff]
