# Supplementary material for: Exposure to pesticides and the risk of hypothyroidism: a systematic review and meta-analysis
Source: BMC Public Health. 2023 Sep 26;23:1867. doi: 10.1186/s12889-023-16721-5 (PMC10523800; doi:10.1186/s12889-023-16721-5)
Supplement: Supplementary file 2 — Additional file 2: Table S1. Quality assessment for reporting observational cohort and cross-sectional, According to the guideline of National Heart, Lung, and Blood Institute (NHLBI). Table S2. Quality assessment for reporting case-control study, According to the guideline of National Heart, Lung, and Blood Institute (NHLBI). Table S3. Subgroup and sensitivity analyses. [file 12889_2023_16721_MOESM2_ESM.docx]

**Table S1.** Quality assessment for reporting observational cohort and cross-sectional, According to the guideline of National Heart, Lung, and Blood Institute (NHLBI)

| **Authors (year)** | **1** | **2** | **3** | **4** | **5** | **6** | **7** | **8** | **9** | **10** | **11** | **12** | **13** | **14** | **Remark** |
| --- | --- | --- | --- | --- | --- | --- | --- | --- | --- | --- | --- | --- | --- | --- | --- |
| Goldner et al.(2010) | Y | Y | Y | Y | N | Y | Y | N | N | N | Y | N | Y | Y |  |
| Goldner et al.(2013) | Y | Y | Y | Y | N | Y | Y | Y | Y | N | Y | N | Y | Y |  |
| Wei et al.(2016) | Y | Y | Y | Y | N | N | N | Y | Y | N | Y | N | NA | Y |  |
| Lerro et al.(2017) | Y | Y | Y | Y | N | Y | Y | Y | Y | Y | Y | N | Y | Y |  |
| Shrestha et al.(2018)^a^ | Y | Y | N | Y | N | Y | Y | Y | Y | N | Y | N | N | Y |  |
| Shrestha et al.(2018)^b^ | Y | Y | Y | Y | N | Y | Y | N | N | N | Y | N | Y | Y |  |
| Londoño et al.(2018) | Y | Y | Y | Y | N | N | N | Y | Y | N | Y | N | NA | Y |  |
| Suhartono et al.(2018) | Y | Y | N | Y | N | N | N | N | Y | N | Y | N | NA | N | Effect estimate was not adjusted for potential confounders and high risk of selection bias |
| Kartini et al.(2018) | Y | N | N | Y | N | N | N | N | N | N | Y | N | NA | N | Effect estimate was not adjusted for potential confounders and high risk of selection bias. Exposure /determinant was not clearly defined and high risk of measurement bias  Exclude from the meta-analysis due to non-specific pesticides exposure |
| Huang et al.(2017) | Y | Y | Y | Y | N | Y | Y | Y | Y | N | Y | N | Y | Y | Exclude from the meta-analysis due to non-specific pesticides exposure |
| Risal et al.(2019) | Y | Y | N | Y | N | N | N | N | N | N | Y | N | NA | N | Effect estimate was not adjusted for potential confounders and high risk of selection bias. Exposure /determinant was not clearly defined and high risk of measurement bias  Exclude from the meta-analysis due to non-specific pesticides exposure |

Y, yes; N, no; CD, cannot determine; NA, not applicable; NR, not reported

**Question**

No.1. Was the research question or objective in this paper clearly stated?

No.2. Was the study population clearly specified and defined?

No.3. Was the participation rate of eligible persons at least 50%?

No.4. Were all the subjects selected or recruited from the same or similar populations (including the same time period)? Were inclusion and exclusion criteria for being in the study prespecified and applied uniformly to all participants?

No.5. Was a sample size justification, power description, or variance and effect estimates provided?

No.6. For the analyses in this paper, were the exposure(s) of interest measured prior to the outcome(s) being measured?

No 7. Was the timeframe sufficient so that one could reasonably expect to see an association between exposure and outcome if it existed?

No.8. For exposures that can vary in amount or level, did the study examine different levels of the exposure as related to the outcome (e.g., categories of exposure, or exposure measured as continuous variable)?

No.9. Were the exposure measures (independent variables) clearly defined, valid, reliable, and implemented consistently across all study participants?

No.10. Was the exposure(s) assessed more than once over time?

No.11. Were the outcome measures (dependent variables) clearly defined, valid, reliable, and implemented consistently across all study participants?

No.12. Were the outcome assessors blinded to the exposure status of participants?

No.13. Was loss to follow-up after baseline 20% or less?

No.14. Were key potential confounding variables measured and adjusted statistically for their impact on the relationship between exposure(s) and outcome(s)?

**Table S2.** Quality assessment for reporting case-control study, According to the guideline of National Heart, Lung, and Blood Institute (NHLBI)

| **Authors (year)** | **1** | **2** | **3** | **4** | **5** | **6** | **7** | **8** | **9** | **10** | **11** | **12** | **Remark** |
| --- | --- | --- | --- | --- | --- | --- | --- | --- | --- | --- | --- | --- | --- |
| Dufour et al.(2020) | Y | Y | N | Y | Y | Y | Y | N | N | Y | N | Y | Unable to confirm that the exposure/risk occurred prior to the development of diagnosed hypothyroidism |

CD, cannot determine; NA, not applicable; NR, not reported

**Question**

1. Was the research question or objective in this paper clearly stated and appropriate?

2. Was the study population clearly specified and defined?

3. Did the authors include a sample size justification?

4. Were controls selected or recruited from the same or similar population that gave rise to the cases (including the same timeframe)?

5. Were the definitions, inclusion and exclusion criteria, algorithms or processes used to identify or select cases and controls valid, reliable, and implemented consistently across all study participants?

6. Were the cases clearly defined and differentiated from controls?

7. If less than 100 percent of eligible cases and/or controls were selected for the study, were the cases and/or controls randomly selected from those eligible?

8. Was there use of concurrent controls?

9. Were the investigators able to confirm that the exposure/risk occurred prior to the development of the condition or event that defined a participant as a case?

10. Were the measures of exposure/risk clearly defined, valid, reliable, and implemented consistently (including the same time period) across all study participants? 11. Were the assessors of exposure/risk blinded to the case or control status of participants?

12. Were key potential confounding variables measured and adjusted statistically in the analyses? If matching was used, did the investigators account for matching during study analysis?

**Table S3.** Subgroup and sensitivity analyses

| **Group of pesticide** | **Condition for analysis** | **Studies included** | **Fixed-effect meta-analysis** | | | | **Random-effects meta-analysis** | | |
| --- | --- | --- | --- | --- | --- | --- | --- | --- | --- |
|  |  |  | **aOR** | **95% CI** | ***p* value** | **aOR** | | **95% CI** | ***p* value** |
| Insecticides-Organochlorines | All | 8 | 1.22 | 1.16 – 1.28 | <0.001 | 1.23 | | 1.14 – 1.33 | <0.001 |
|  | *Study design* | | | | | | | | |
|  | Cohort studies | 5 | 1.19 | 1.34 – 1.25 | <0.001 | 1.18 | | 1.10 – 1.26 | <0.001 |
|  | Cross-sectional studies | 2 | 1.77 | 1.42 – 2.20 | <0.001 | 1.92 | | 1.42 – 2.60 | <0.001 |
|  | *Type of exposure* | | | | | | | | |
|  | Occupational | 6 | 1.21 | 1.16 – 1.27 | <0.001 | 1.22 | | 1.14 – 1.32 | <0.001 |
|  | Environmental | 2 | 4.31 | 1.34 – 13.89 | 0.014 | 4.01 | | 0.88 – 18.23 | 0.072 |
|  | *Type of outcome* | | | | | | | | |
|  | Any hypothyroidism | 4 | 1.19 | 1.13 – 1.25 | <0.001 | 1.18 | | 1.10 – 1.25 | <0.001 |
|  | Overt hypothyroidism | 3 | 1.83 | 1.08 – 3.12 | 0.026 | 2.09 | | 0.91 – 4.78 | 0.082 |
|  | Subclinical hypothyroidism | 2 | 1.77 | 1.41 – 2.21 | <0.001 | 1.81 | | 1.41 – 2.32 | <0.001 |
|  | *Exclusion of a single study* | | | | | | | | |
|  | Goldner et al.[27] | 7 | 1.21 | 1.15 – 1.27 | <0.001 | 1.23 | | 1.13 – 1.34 | <0.001 |
|  | Goldner et al.[28] | 7 | 1.19 | 1.13 – 1.27 | <0.001 | 1.24 | | 1.12 – 1.37 | <0.001 |
|  | Wei et al.[29] | 7 | 1.22 | 1.16 – 1.27 | <0.001 | 1.23 | | 1.14 – 1.32 | <0.001 |
|  | Lerro et al.[30] | 7 | 1.21 | 1.16 – 1.27 | <0.001 | 1.22 | | 1.14 – 1.32 | <0.001 |
|  | Shresta et al.[20] | 7 | 1.31 | 1.22 – 1.40 | <0.001 | 1.35 | | 1.22 – 1.48 | <0.001 |
|  | Shresta et al.[21] | 7 | 1.22 | 1.16 – 1.28 | <0.001 | 1.23 | | 1.14 – 1.33 | <0.001 |
|  | Londoño et al.[31] | 7 | 1.20 | 1.14 – 1.26 | <0.001 | 1.18 | | 1.10 – 1.27 | <0.001 |
|  | Dufour et al.[32] | 7 | 1.21 | 1.16 – 1.27 | <0.001 | 1.23 | | 1.14 – 1.32 | <0.001 |
| Insecticides-Organophosphates | All | 6 | 1.12 | 1.08 – 1.16 | <0.001 | 1.12 | | 1.07 – 1,17 | <0.001 |
|  | *Study design* | | | | | | | | |
|  | Cohort studies | 5 | 1.12 | 1.08 – 1.16 | <0.001 | 1.12 | | 1.07 – 1.17 | <0.001 |
|  | *Type of exposure* | | | | | | | | |
|  | Occupational | 5 | 1.12 | 1.08 – 1.16 | <0.001 | 1.12 | | 1.07 – 1.17 | <0.001 |
|  | *Type of outcome* | | | | | | | | |
|  | Any hypothyroidism | 4 | 1.12 | 1.08 – 1.16 | <0.001 | 1.12 | | 1.07 – 1.17 | <0.001 |
|  | Subclinical hypothyroidism | 2 | 1.17 | 0.87 – 1.58 | 0.294 | 1.24 | | 0.84 – 1.82 | 0.276 |
|  | *Exclusion of a single study* | | | | | | | | |
|  | Goldner et al.[27] | 5 | 1.14 | 1.09 – 1.18 | <0.001 | 1.14 | | 1.08 – 1.20 | <0.001 |
|  | Goldner et al.[28] | 5 | 1.11 | 1.07 – 1.16 | <0.001 | 1.10 | | 1.04 – 1.17 | 0.002 |
|  | Lerro et al.[30] | 5 | 1.12 | 1.08 – 1.16 | <0.001 | 1.12 | | 1.07 – 1.17 | <0.001 |
|  | Shresta et al.[20] | 5 | 1.10 | 1.05 – 1.15 | <0.001 | 1.09 | | 1.03 – 1.16 | 0.002 |
|  | Shresta et al.[21] | 5 | 1.13 | 1.09 – 1.18 | <0.001 | 1.13 | | 1.08 – 1.18 | <0.001 |
|  | Suhartono et al.[19] | 5 | 1.12 | 1.08 – 1.16 | <0.001 | 1.12 | | 1.07 – 1.17 | <0.001 |
| Insecticides-Carbamates | All | 5 | 1.05 | 0.99 – 1.11 | 0.067 | 1.05 | | 0.99 – 1.12 | 0.120 |
|  | *Type of outcome* | | | | | | | | |
|  | Any hypothyroidism | 4 | 1.05 | 0.99 – 1.10 | 0.080 | 1.05 | | 0.98 - 1.12 | 0.147 |
|  | Subclinical hypothyroidism | 1 | 1.29 | 0.74 – 2.26 | 0.368 | 1.25 | | 0.64 – 2.42 | 0.512 |
|  | *Exclusion of a single study* | | | | | | | | |
|  | Goldner et al.[27] | 4 | 1.08 | 1.01 – 1.15 | 0.024 | 1.07 | | 0.98 – 1.17 | 0.113 |
|  | Goldner et al.[28] | 4 | 1.03 | 0.97 – 1.09 | 0.331 | 1.29 | | 0.96 – 1.09 | 0.365 |
|  | Lerro et al.[30] | 4 | 1.05 | 0.99 – 1.11 | 0.080 | 1.05 | | 0.98 – 1.12 | 0.147 |
|  | Shresta et al.[20] | 4 | 1.04 | 0.98 – 1.11 | 0.177 | 1.04 | | 0.98 – 1.11 | 0.177 |
|  | Shresta et al.[21] | 4 | 1.06 | 1.00 – 1.12 | 0.057 | 1.06 | | 0.98 – 1.15 | 0.147 |
| Insecticides-Pyrethroids | All | 4 | 1.15 | 1.03 – 1.28 | 0.012 | 1.13 | | 0.99 – 1.29 | 0.116 |
|  | *Type of outcome* | | | | | | | | |
|  | Any hypothyroidism | 3 | 1.15 | 1.03 – 1.28 | 0.013 | 1.13 | | 0.97 – 1.30 | 0.064 |
|  | *Exclusion of a single study* | | | | | | | | |
|  | Goldner et al.[27] | 3 | 1.18 | 1.06 – 1.32 | 0.003 | 1.18 | | 1.06 – 1.32 | 0.003 |
|  | Goldner et al.[28] | 3 | 1.14 | 1.00 – 1.30 | 0.054 | 1.07 | | 0.85 – 1.36 | 0.569 |
|  | Lerro et al.[30] | 3 | 1.15 | 1.03 – 1.28 | 0.013 | 1.13 | | 0.97 – 1.30 | 0.116 |
|  | Shresta et al.[20] | 3 | 1.08 | 0.91 – 1.28 | 0.366 | 1.03 | | 0.79 – 1.34 | 0.831 |
| Herbicides | All | 5 | 1.05 | 1.03 – 1.08 | 0.007 | 1.06 | | 1.02 – 1.10 | 0.003 |
|  | *Type of outcome* | | | | | | | | |
|  | Any hypothyroidism | 4 | 1.05 | 1.02 – 1.08 | <0.001 | 1.05 | | 1.01 – 1.09 | 0.011 |
|  | Subclinical hypothyroidism | 1 | 1.30 | 1.07 – 1.57 | <0.001 | 1.30 | | 1.07 – 1.57 | 0.007 |
|  | *Exclusion of a single study* | | | | | | | | |
|  | Goldner et al.[27] | 4 | 1.07 | 1.04 – 1.10 | <0.001 | 1.08 | | 1.04 – 1.12 | <0.001 |
|  | Goldner et al.[28] | 4 | 1.03 | 1.00 – 1.06 | 0.037 | 1.03 | | 0.99 – 1.08 | 0.140 |
|  | Lerro et al.[30] | 4 | 1.05 | 1.02 – 1.08 | <0.001 | 1.05 | | 1.01 – 1.09 | 0.011 |
|  | Shresta et al.[20] | 4 | 1.06 | 1.02 – 1.09 | 0.001 | 1.06 | | 1.00 – 1.11 | 0.034 |
|  | Shresta et al.[21] | 4 | 1.06 | 1.03 – 1.09 | <0.001 | 1.07 | | 1.03 – 1.11 | 0.001 |
| Fungicides | All | 5 | 1.05 | 0.97 – 1.14 | 0.211 | 1.15 | | 0.97 – 1.36 | 0.112 |
|  | *Type of outcome* | | | | | | | | |
|  | Any hypothyroidism | 4 | 1.05 | 0.97 – 1.13 | 0.247 | 1.14 | | 0.96 – 1.36 | 0.132 |
|  | Subclinical hypothyroidism | 1 | 1.32 | 0.67 – 2.52 | 0.394 | 1.16 | | 0.38 – 3.52 | 0.797 |
|  | *Exclusion of a single study* | | | | | | | | |
|  | Goldner et al.[27] | 4 | 0.97 | 0.89 – 1.05 | 0.440 | 0.99 | | 0.86 – 1.14 | 0.872 |
|  | Goldner et al.[28] | 4 | 1.09 | 1.00 – 1.20 | 0.056 | 1.26 | | 1.02 – 1.55 | 0.032 |
|  | Lerro et al.[30] | 4 | 1.05 | 0.97 – 1.13 | 0.247 | 1.14 | | 0.96 – 1.36 | 0.132 |
|  | Shresta et al.[20] | 4 | 1.21 | 1.09 – 1.36 | 0.001 | 1.29 | | 0.99 – 1.68 | 0.055 |
|  | Shresta et al.[21] | 4 | 1.03 | 0.95 – 1.11 | 0.531 | 1.11 | | 0.94 – 1.31 | 0.216 |
| Fumigants | All | 4 | 1.01 | 0.90 – 1.12 | 0.931 | 1.01 | | 0.90 – 1.12 | 0.931 |
|  | *Type of outcome* | | | | | | | | |
|  | Any hypothyroidism | 4 | 1.01 | 0.91 – 1.13 | 0.870 | 1.01 | | 0.90 – 1.13 | 0.851 |
|  | Subclinical hypothyroidism | 1 | 0.71 | 0.27 – 1.87 | 0.494 | 0.72 | | 0.27 – 1.87 | 0.494 |
|  | *Exclusion of a single study* | | | | | | | | |
|  | Goldner et al.[27] | 3 | 0.96 | 0.86 -1.08 | 0.514 | 0.96 | | 0.86 -1.08 | 0.514 |
|  | Goldner et al.[28] | 3 | 1.03 | 0.90 – 1.18 | 0.666 | 1.03 | | 0.90 – 1.19 | 0.646 |
|  | Lerro et al.[30] | 3 | 1.01 | 0.91 – 1.13 | 0.870 | 1.01 | | 0.90 – 1.13 | 0.851 |
|  | Shresta et al.[20] | 3 | 1.05 | 0.89 – 1.23 | 0.587 | 1.06 | | 0.87 – 1.29 | 0.552 |
